# Defensive Medicine among Obstetricians and Gynecologists in Tertiary Hospitals

**DOI:** 10.1371/journal.pone.0057108

**Published:** 2013-03-06

**Authors:** Elad Asher, Shay Dvir, Daniel S. Seidman, Sari Greenberg-Dotan, Alon Kedem, Boaz Sheizaf, Haim Reuveni

**Affiliations:** 1 Sheba Medical Center, Tel-Hashomer, Ramat Gan, Israel; 2 Sackler School of Medicine, Tel-Aviv University, Tel-Aviv, Israel; 3 Division of Obstetrics and Gynecology, Sheba Medical Center, Tel-Hashomer, Ramat Gan, Israel; 4 Ben-Gurion University of the Negev, Beer-Sheva, Israel; 5 Division of Obstetrics and Gynecology, Soroka Medical Center, Beer-Sheva, Israel; Tehran University of Medical Sciences, Iran (Islamic Republic of)

## Abstract

**Objective:**

To describe the daily work practice under the threat of defensive medicine among obstetricians and gynecologists.

**Study Design:**

A prospective cross-sectional survey of obstetricians and gynecologists working at tertiary medical centers in Israel.

**Results:**

Among the 117 obstetricians and gynecologists who participated in the survey, representing 10% of the obstetricians and gynecologists registered by the Israel Medical Association, 113 (97%) felt that their daily work practice is influenced by concern about being sued for medical negligence and not only by genuine medical considerations. As a result, 102 (87%) physicians are more likely to offer the cesarean section option, even in the absence of a clear medical indication, 70 (60%) follow court rulings concerning medical practices, and 85 (73%) physicians mentioned that discussions about medical negligence court rulings are included in their departments' meetings.

**Conclusions:**

Defensive medicine is a well-embedded phenomenon affecting the medical decision process of obstetricians and gynecologists.

## Introduction

Mistakes are an inevitable part of medical practice, making medical negligence claims a common event [1]. Patient dissatisfaction and doctors' interpersonal relations with their patients are among the main reasons for medical negligence claims [2]–[4].

Defensive medicine is defined as “medical actions, performed mainly in order to refrain from being sued, rather than actually aiding the patient.” [5] Some would claim that it is a legitimate phenomenon, while others consider it immoral [6].

The costs (direct and indirect) of defensive medicine to the American health care system are estimated to be around $50 billion a year [7], [8]. A genuine difficulty exists when trying to identify and quantify the extent of defensive medicine practices. This is partially because there is a grey area between proper and overly self-protective treatment. Also, it may be difficult to recognize medical actions that are more likely to result in legal action.

The perceived threat of malpractice among physicians may boil down to three factors: the risk, the probability, and the size of payment of a claim. The large number of claims that do not lead to payment may add to the perceived malpractice risk (e.g., stress, added work, reputation damage) [9]. In fact, nearly 40% of claims are not associated with medical errors, but they account for 16% of total liability costs in the system [10]. About 5–7.4% of physicians [11] faced a malpractice claim during the year prior to the survey. Gynecology alone had the 12th highest average annual proportion of physicians with a claim, with the highest payment rate (>38%). Obstetrics and general surgery are regarded as high-risk specialties [11].

The general atmosphere of judicialization within the medical environment is mostly noticeable in the gynecology and obstetrics fields, in which both young, healthy women and the fetus are at risk [12]. In Pennsylvania, the use of medical procedures correlated with the trust the doctors had in their policies [13] and did not correlate with either the potential severity of the finding or with the degree of certainty of the need for treatment. The fear of being sued directly and indirectly increases the rate of cesarean sections (CSs) [14]. It was estimated that among 27.6% of the CSs that were performed, 6.6% were done out of legal concerns, rather than necessity [14].

In Israel 4200–4500 medical claims are filed annually. The average compensation per patient was $62,000 in 2008–2009, compared with $22,000 only 5 years earlier. During 1985–1998 obstetrics and emergency medicine departments were on the top of the list of medical claims [1]. The extent of compensation paid under court rulings in obstetrics and gynecology (data from financial newspaper *Calcalist*, September 2009) is constantly increasing to a current average of $250,000 per claim. As a result, the rising cost of malpractice insurance in obstetrics and genecology has led to a reality where doctors may refrain from treating high risk patients [12], [13].

In this study we examined the extent and possible effect of the defensive medicine phenomenon on medical decision making among OB-GYN physicians. We assumed that defensive medicine may strongly influence affect their clinical judgments in daily practice.

## Materials and Methods

### Type

A prospective cross-sectional survey.

### Setting

In January 1995, an obligatory National Health Insurance (NHI) law went into effect in Israel, securing the right of every resident to a well-defined list of health services. The six tertiary medical centers in Israel [Soroka University Medical Center (996 beds), Hadassah Medical Centers (887 beds), Tel-Aviv Sourasky Medical Center (1050 beds), Rabin Medical Center (1094 beds), The Sheba Medical Center (1430 beds), and Rambam Medical Center (898 beds)] are academic centers affiliated with the four medical schools in Israel. OB-GYN physicians in Israel practice medicine as in most advanced Organisation for Economic Co-operation and Development (OECD) countries.

### Study population

Included board certified physicians and residents from the OB-GYN departments. We excluded visiting OB-GYNs who do not practice at these tertiary hospitals.

The dependent variable was defined as “the existence and extent of defensive medicine among OB-GYNs”.

The independent variables included: demographic data (age, gender, institution of graduation), professional data (specialists and residents), exposure to medical negligence court rulings, concern about being sued, and daily work habits, as they are perceived by the doctors themselves.

### The questionnaire

Confidential questionnaire was developed by a team of board certified physicians, lawyers, and specialists in health systems management and ethics. For validation, a preliminary study was conducted among 10 specialist physicians. After comments, the final questionnaire was approved. None of the 10 physicians used in the validation participated in the study. The questionnaire was then given to OB-GYN physicians in the tertiary hospitals mentioned above during their morning meetings. The questionnaire consisted of 18 questions including:

- personal details (gender, age, and professional status)
- awareness and habits related to defensive medicine
- physician-patient relationships influenced by the concern for legal demands

a case description: “A woman arrives at your department with an estimated fetus weight of 4.2 kg. She is generally healthy, has no diabetes or other chronic diseases, and has no history of babies born overweight. Given the above, would you perform a CS or a vaginal delivery?” The responders had to answer the question given two scenarios: the first takes place in their department, the second takes place in a remote place, where the concern for legal demands is hardly an issue. According to the American congress of obstetrics and gynecology (ACOG) and the National Institute for Health and Clinical Excellence there are only several cases in which CS should be consider (previous cesarean delivery; mechanical obstruction that prevents or complicates vaginal delivery, such as large fibroids or a pelvic fracture; large infant >4.5 kg/9 lbs, especially if the mother has diabetes; an active infection, such as herpes or HIV; multiple gestation - twins, triplets, or more; cervical cancer; increased infant risk of bleeding; the placenta is covering the cervix- placenta previa) [15], [16]. None of these scenarios were included in the case aforementioned, hence there was no indication for CS but for vaginal delivery. According to the academic medical center at Ben-Gurion University, ethical approval for this study was deemed unnecessary since this study was a physician's survey and no patient was included. Every physician verbally consented to participate in the survey when filling the questionnaire.

### Survey content and main outcome measures

All Physicians were asked about their concerns regarding malpractice liability and whether it caused them to act in each of four forms of “assurance” behavior: (1) order more tests than medically indicated; (2) suggest invasive procedures against professional judgment; (3) fill in more forms due to fear from litigation; (4) giving too much information to the patients in a way that might create confusion and limit their ability to obtain an informed consent. Physicians who reported engaging defensive medicine practices were then asked in an open question to describe their most recent act of defensive medicine.

### Statistical analyses

We used descriptive statistics as percent distribution, mean and standard deviation. Association between stages of residency, gender and age versus daily work habits that define defensive medicine, were analyzed using Chi- square test.A multivariate analysis was performed in order to describe characteristics of physicians practicing defensive medicine. Predictor's variables were: age, gender, and professional status. All tests were two sided, and values of p<0.05 were considered statistically significant, using SPSS 15 (SPSS Inc., Chicago, IL).

## Results

### Study population

(Table S1) included 117 (response rate of 80%) OB-GYN physicians who represent 10% of all OB-GYN physicians listed by the Israel Medical Association (IMA). Of those, 95 (81%) were men, with a mean age of 50 years. Eighty-eight (75%) were board certified specialists in the fields of OB-GYN; 29 (25%) were still residents.

### Working atmosphere

Seventy (60%) physicians mentioned they read court rulings concerning medical practices, 85 (73%) physicians mentioned that discussions about medical negligence court rulings are held in their department meetings, and 70 (60%) physicians mentioned that court rulings regarding ERB palsy (a paralysis of the arm caused by injury to the upper group of the arm's main nerves, specifically the severing of the upper trunk C5–C6 nerves) caused during delivery affect them strongly/very strongly when choosing the mode of delivery for a 4.2 kg fetus. One hundred and eleven (95%) said they are influenced, to some extent, by these court rulings (Table S2).

**Figure 1:**
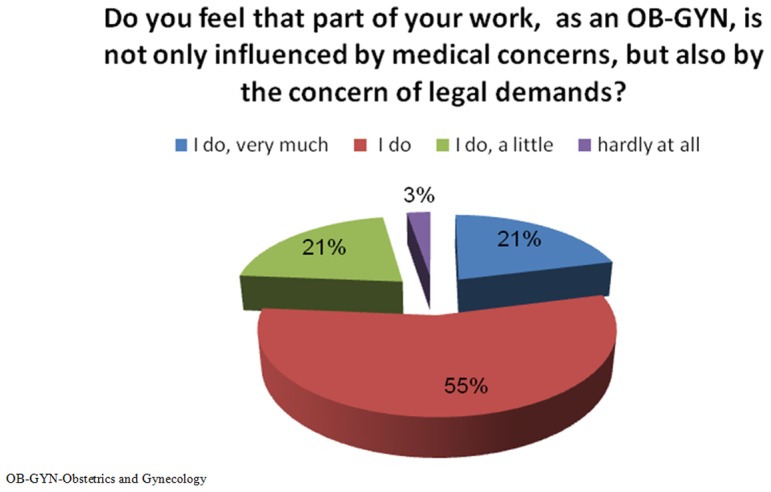
Concern regarding legal claims in the work of the OB-GYNs.

One hundred and eleven (95%) physicians were concerned with facing a legal claim (Figure 1). One hundred and one (86%) physicians stated they insisted on having the patient sign an informed consent form for any procedure, “no matter what”, even if they were not required to do so by the hospital before a certain procedure. On the other hand, 49 (42%) physicians admitted giving too much information (e.g., risks, both major and minor) to the patients in a way that might create confusion and limit their ability to obtain an informed consent.

### Cesarean section vs. vaginal delivery

One hundred and two (87%) physicians, who were concerned about a legal claim in case of injury to the fetus during delivery, tended to offer the CS option more often, even in the absence of a clear medical indication.

Ninety-four (80%) physicians thought that the delivery guidelines (vaginal delivery vs. CS) [15] are influenced by medical considerations, and precaution standards set in accordance with Israeli court rulings. Ninety-three (79%) physicians document, in each and every childbirth, all considerations regarding the mode of delivery.

In response to the last question, regarding the performance of CS under two different scenarios: 40 (34%) physicians would perform a CS in their departments, whereas only 24 (21%) physicians would do so had they been working in a remote place (*p*<0.0001). Moreover, 78% percent of OB-GYN physicians were concerned during their daily work about a potential legal claim.

In a multivariable analysis no significant differences were found between the physician subgroups (age, gender, and professional status) regarding the defensive doctor features.

## Discussion

This study shows that defensive medicine is deeply rooted in the everyday work of OB-GYNs in tertiary hospitals in Israel. It also shows that a general atmosphere in the public and the media that is critical of the OB-GYN physicians' work motivates them to practice defensive medicine in order to protect themselves from being sued. Defensive medicine is highly prevalent in all age groups and is similar regardless of gender and professional status, specialists or residents.

Our study group included 117 OB-GYN physicians from all tertiary hospitals in Israel. These physicians provide the most complex and high quality care, as in most advanced industrial countries (OECD) [17]. The Israeli health system is universal and participation in a medical insurance plan is compulsory. All Israeli citizens are entitled to a Uniform Benefits Package, regardless of their financial means.

Among the Israeli OB-GYN physicians, 95% were concerned about being sued. They are not unique in this regard. Among American physicians specializing in fields of medicine in which litigation is frequent (such as surgeons, radiologists, and OB-GYNs), 93% reported practicing defensive medicine. Accordingly, the cost of defensive medicine to the American health system is estimated at $50 billion a year [13].

Sixty percent of OB-GYN physicians routinely read court rulings regarding medical practice, and 73% declared that discussions of medical negligence court rulings are part of morning meetings in their departments. These findings probably affect daily practice when choosing a mode of care. In a liberal country such as Israel, the media plays a major role in propagating the advantages and disadvantages of certain medical procedures, as well as exposing cases of medical mishaps and court rulings regarding these matters. Moreover, the attitudes of the society, including the patients, the media, and the court, reflect intolerance to risk [18].

Eighty-seven percent of OB-GYN physicians would offer an unnecessary procedure, such as CS, to avoid a possible legal claim. Previous studies have already demonstrated the high prevalence of elective CSs in the Western world. The primary cause of these elective CSs, according to one study, was maternal preference and the OB-GYN physicians will to respond to that preference [19]. Another study emphasized the safe image CSs have in comparison to the possible substantial morbidity of vaginal delivery and the intolerance of our society for risk [18]. In this study, OB-GYN physicians reaffirmed the conclusion raised by several other studies, according to which, *many* elective CSs are due to defensive medicine [20], [21]. Hence, this underlines the role of defensive medicine as a major factor to consider when analyzing the overuse of CSs in developed countries.

Eighty percent of OB-GYN physicians say that the current guidelines are already influenced by legal precautions. Guidelines are consensus statements, systematically developed on the basis of evidence to promote the effectiveness and safety of healthcare delivery. Their use provides doctors with greater certainty as to what is expected of them by law [22]. Israeli OB-GYN physicians often work according to American guidelines. The Israeli Society for Obstetrics and Gynecology (ISOG) is the official organization representing the Obstetricians and Gynecologists in Israel and is part of the Israeli Medical Association. Although the ISOG publishes position papers from time to time the OB-GYN physicians in Israel work according to the ACOG guidelines. Most of the OB-GYN physicians who we surveyed declared that by practicing these guidelines they perform defensive medicine. The actual meaning of the above is that OB-GYN physicians feel that working according to current regulations is first not enough to refrain from claims, and second not necessarily what is medically right. It therefore calls for measures to be taken in order to minimize the gap between what is perceived as “the right medical actions” and the actions required to follow the regulations.

Now, what is wrong with the use of defensive medicine by OB-GYN physicians? One of the main problems is the financial cost to the system. Good examples are the unnecessary referral to specialists, the overuse of imaging techniques (e.g., ultrasonography), and the non-essential orders for biopsy [13]. Another problem is the abstention of doctors from fields with known risk of legal demands: both the desire to refrain from these demands and the resulting rising costs of insurance policies, creating a shortage of doctors in these fields in a way that may harm women's health [12], [13].

In a recent published Israeli national survey [23] which was the first nationwide survey regarding the practice of defensive medicine performed among representative sample of 877 board certified expert physicians from eight medical disciplines, several steps to decrease the presence of defensive medicine in their everyday work were offered: medical debriefings of exceptional events, greater transparency to the patient, expectations adaptation with patients, diminution of financial considerations when treating a patient, limiting the compensation fees given to settle medical negligence cases, and finally, new legislation that better protects the doctors' rights.

Several system models have been offered in the literature in the past to cope with the defensive medicine phenomenon: [22]–[24]

1. *The “alternative dispute” resolution* – a committee discussion, outside the court, that is less formal, less public, and less expensive. Still, this solution is only partial, because doctors' concern is not only the court, but also any investigative procedure. 2. *The “comprehensive responsibility” solution* – the doctor would no longer hold responsibility for legal claims. Instead, this would become the medical center's responsibility. This may reduce anxiety and concern on the doctor's part, as well as allowing the medical centers to be more fastidious about the doctors they choose, making sure to let them understand perfectly the protocols and developing a risk management system. Nevertheless, there is a chance that the organization would limit the doctors' thinking autonomy under more strict regulations. 3. *The “selective no*-*fault” solution* – medical negligence victims would be entitled to compensation fees, regardless of who is responsible. This solution does not refer to an automatic compensation system, but to a selective system that defines in advance a list of preventable events that might damage the patient. This solution removes the psychological concern of investigations and legal claims. Still, this would not necessarily be applied to all cases, and it is still possible that some cases would be transferred to the courts. 4. *Clinical practice guidelines* – written guidelines are becoming more prevalent in all fields of medicine, both in order to create better, more uniform medicine and to avoid medical negligence legal demands. Still, guidelines have limitations and represent a generalization of empirical evidence that may not always be applicable to individual patients due to the need for the exercise of clinical judgment.

In informal conversations with the gynecologists, the problems we found were typical to countries like the United States and elsewhere where a third party (insurance company) can pay for claims. In addition, we think that further discussion and research on the subject will flood the problem to professional and public discussion towards the direct impact of public costs due to doctors' insurance and unnecessary tests. Moreover, there are not enough studies and research methods that examine the outcomes of defensive medicine in terms of impact on patients' and caregivers quality of life as well as in terms of cost effectiveness [9], [10], [23], [25]–[26].

### Study limitations

Objective methods for measuring defensive medicine are very difficult [5], [23], [27] because distinctions between inappropriate and appropriate care are not always clear in many clinical situations [13], [26]. It is also difficult to identify the difference between liability-related motivators and other factors that influence clinical decision making. Another limitation is that physician self-reports of defensive medicine may be biased, and may lead physicians to overstate the frequency of performing defensive medicine. In contrast, unconscious defensive medicine is not reported by physicians but it is also widely practiced [25], [27].

### Summary and conclusion

- This study strongly suggests that defensive medicine is deeply rooted in the everyday work of OB-GYN physicians. This may be relevant to other health systems where the prevalence of physician litigation is increasing.
- The overuse of CSs is probably the iceberg phenomenon of other clinical decisions associated with the practice of defensive medicine [28]. One of the most frequent daily practice of defensive medicine is performing more unnecessary tests and referring more patients to consultants and hospitalization [23].

## Supporting Information

Table S1 Physicians' Characteristics.
(DOC)

Table S2 Defensive medicine in daily practice (n = 117 physicians).
(DOCX)

Figure S1 (TIF)
