# Supplementary material for: Defensive Medicine among Obstetricians and Gynecologists in Tertiary Hospitals
Source: PLoS One. 2013 Mar 6;8(3):e57108. doi: 10.1371/journal.pone.0057108 (PMC3590209; doi:10.1371/journal.pone.0057108)
Supplement: Table S1 — Physicians' Characteristics. (DOC) [file pone.0057108.s001.doc]

Table S1: Physicians’ Characteristics.

| **Characteristics** | **No. (%) of Physicians (N=117)** |
| --- | --- |
| **Age** |  |
| 18–29 | 2 (1.7%) |
| 30–39 | 44 (37.6%) |
| 40–54 | 32 (27.4%) |
| >55 | 39 (33.3%) |
|  |  |
| **Gender** |  |
| Male | 95 (81%) |
| Female | 22 (19%) |
|  |  |
| **Physicians professional position** |  |
| OB-GYB Board certified physicians | 88 (75%) |
| OB-GYN residents | 29 (25%) |
