# Supplementary material for: Defensive Medicine among Obstetricians and Gynecologists in Tertiary Hospitals
Source: PLoS One. 2013 Mar 6;8(3):e57108. doi: 10.1371/journal.pone.0057108 (PMC3590209; doi:10.1371/journal.pone.0057108)
Supplement: Table S2 — Defensive medicine in daily practice (n = 117 physicians). (DOCX) [file pone.0057108.s002.docx]

Table S2: Defensive medicine in daily practice (n = 117 physicians).

| **Cesarean section vs. vaginal delivery** |  |
| --- | --- |
| Tend to offer the CS option more often, even in the absence of a  clear medical indication due to concerns about a legal claim | 102 (87%) |
| Document, in each and every childbirth, all considerations regarding  the mode of delivery | 93 (79%) |
| **Working atmosphere** |  |
| Read court rulings concerning medical practices | 70 (60%) |
| Discussions about medical negligence court rulings are held in  department’s meetings | 85 (73%) |
| Concern with facing a legal claim | 111 (95%) |
| Insist on having the patient sign an informed consent form for any  procedure, even if it’s not a hospital requirement | 101 (86%) |
| Giving too much information (e.g., risks, both major and minor) to the  patients in a way that might create patient confusion | 49 (42%) |
