# Supplementary figures and images for: Defensive Medicine among Obstetricians and Gynecologists in Tertiary Hospitals
Source: PLoS One. 2013 Mar 6;8(3):e57108. doi: 10.1371/journal.pone.0057108 (PMC3590209; doi:10.1371/journal.pone.0057108)

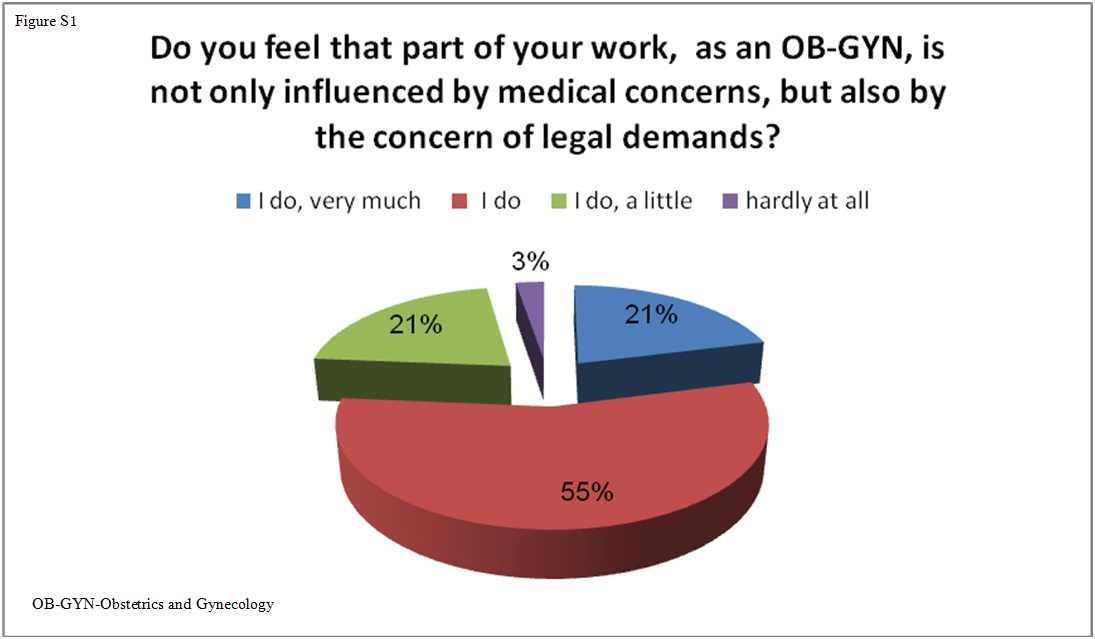

Supplement: Figure S1 — (TIF) [file pone.0057108.s003.tif]
